# Methylation Profiles Differ According to Clinical Characteristics in Well-Differentiated Neuroendocrine Tumors of the Lung

**DOI:** 10.1007/s12022-025-09847-2

**Published:** 2025-03-12

**Authors:** Philipp Melhorn, Erwin Tomasich, Alissa Blessing, Luka Brcic, Angelika Kogler, Alexander Draschl, Peter Mazal, Anna Sophie Berghoff, Markus Raderer, Matthias Preusser, Gerwin Heller, Barbara Kiesewetter

**Affiliations:** 1 Division of Oncology, Department of Medicine I, Medical University of Vienna, Vienna, Austria; 2 Department of Pathology, Medical University of Vienna, Vienna, Austria; 3 Diagnostic and Research Institute of Pathology, Medical University of Graz, Graz, Austria

**Keywords:** Lung neuroendocrine tumors, Lung carcinoids, Methylation, Epigenetics, Biostatistics

## Abstract

Neuroendocrine tumors (NET) of the lung constitute a rare entity of primary lung malignancies that often exhibit an indolent clinical course. Epigenetics-related differences have been described previously for lung NET, but the clinical significance remains unclear. In this study, we performed genome-wide methylation analysis using the Infinium MethylationEPIC BeadChip technology on FFPE tissues from lung NET treated at two academic centers. We aimed to investigate the methylation profiles of known prognostic subgroups. In total, 54 tissue samples from primary lung NET were analyzed, of which 37 were typical carcinoids (TC) and 17 atypical carcinoids (AC). Overall, 25/53 patients (47.2%) developed metastases throughout the disease course, 14/26 (53.8%) had a positive somatostatin receptor (SSTR) scan, and 7/28 patients (25.0%) had documented endocrine activity. Analysis of the DNA methylation data showed substantial differences between TC and AC samples and revealed three distinct clusters (C1–C3): C3 (*n* = 29) with 100% TC and 89.7% non-metastasized, C2 (*n* = 22) with 63.6% AC and 95.5% metastasized, and C1 with three AC samples (2/3 metastasized). In subgroup analyses, distinct methylation patterns were observed based on histology, metastases, SSTR status, and endocrine activity. In the functional gene classification, the genes affected by differential methylation were mainly involved in cell signaling. DNA methylation could potentially aid in the diagnostic process of lung NET. The differences in methylation observed with respect to clinical features like SSTR expression and endocrine activity could translate into improved management of lung NET.

**Supplementary Information:**

The online version contains supplementary material available at 10.1007/s12022-025-09847-2.

## Introduction

Neuroendocrine neoplasms (NEN) of the lung comprise a spectrum of malignancies that share neuroendocrine features in histology but may have a diverse tumor biology, ranging from indolent to highly aggressive [1]. The 2022 WHO classification categorizes pulmonary NEN based on differentiation and grade, distinguishing well-differentiated neuroendocrine tumors (NET) from poorly-differentiated neuroendocrine carcinoma (NEC) like large-cell NEC and small-cell lung carcinoma [2]. Lung NET include typical carcinoids (TC, < 2 mitoses/2 mm^2^ and no necrosis) and atypical carcinoids (AC, 2–10 mitoses/2 mm^2^ and/or necrosis) and account for 1–2% of all primary lung tumors (AC represent only 10% of lung NET) [1, 2].

The clinical presentation of lung NET can be asymptomatic to nonspecific, but a certain subset of patients may experience characteristic hormonal symptoms (e.g., Cushing’s syndrome or carcinoid syndrome) [3–5]. The prognosis is generally favorable, with 10-year disease-specific survival of about 60% for TC and 20% for AC in stage IV [6]. Other known prognostic factors, except nodal status and differentiation, are Ki-67 index, age, surgery, or radiation of the primary site and SSTR status [7–9]. The therapeutic armamentarium for advanced lung NET is limited, and everolimus is the only FDA/EMA-approved compound for antiproliferative use in lung NET to date [10]. According to the current European guidelines, further treatment strategies include somatostatin analogs (SSA), temozolomide-based chemotherapy, peptide receptor radionuclide therapy (PRRT), platinum-based chemotherapy, and interferon-α [10, 11].

Pulmonary carcinoids frequently have mutations in histone-modification and chromatin-remodeling genes, and there are distinct differences between carcinoids and carcinomas, as *MEN1* alterations are exclusive to carcinoids, and *TP53* and *RB1* mutations enriched in carcinomas [12, 13]. In terms of epigenetics, two research groups have conducted methylation analyses in lung NET, each describing three distinct clusters that were enriched for specific pathologic features such as *MEN1* mutation or a certain histologic subtype [14, 15]. Nevertheless, neither study provided substantial clinical or outcome data, so the clinical significance of methylation in lung NET remains unclear.

Thus, the main objective of this study was to elucidate the potential correlation of clinical characteristics and methylation patterns in lung NET. To that objective, we have collected a sizeable and clinically well-characterized cohort of pulmonary carcinoids from two tertiary referral centers, performed genome-wide methylome profiling of over 850,000 CpG sites using the Illumina MethylationEPIC BeadChip, and then correlated epigenetic results with clinical features, i.e., histologic subtype, metastatic disease, SSTR status, and endocrine activity.

## Methods

### Inclusion Criteria and Data Collection

This study included histologically verified lung NET patients from two academic centers (Medical University of Vienna and Medical University of Graz) who were diagnosed with either TC or AC and had sufficient formalin-fixed paraffin-embedded (FFPE) tissue from the primary tumor or metastases available for methylation analysis (one sample per patient). At both sites, clinical data were collected via retrospective chart review, including basic clinical characteristics (sex, age, date of diagnosis, and Eastern Cooperative Oncology Group (ECOG) status), histologic characteristics (grading, Ki-67 index, mitotic count, and SSTR2/5 expression), disease characteristics (primary localization, tumor stage, metastases, endocrine activity, and functional imaging), and treatment information (surgery, systemic therapy lines, response, progression-free survival, overall survival, and death if applicable). This study had received approval by the Ethics Committee of the Medical University of Vienna (EK no.: 1918/2020).

### DNA Extraction from FFPE Tissue

FFPE tissue blocks from selected patients were evaluated by NET reference pathologists (P.M., L.B.) based on the corresponding hematoxylin–eosin (H&E) staining, and regions with the highest tumor cell content were selected. Tumor tissues were separated from the block by specific biopsy punching needles (Ø 1 mm) or macro-dissection depending on the presentation of tumor tissue. Genomic DNA was isolated using the Maxwell FFPE Plus DNA Kit (Promega, Madison, Wisconsin, USA) according to the manufacturer’s instruction. The Infinium HD FFPE Restore Kit (Illumina, San Diego, California, USA) was used to repair degraded DNA to improve downstream amplification. Bisulfite treatment was performed using the EpiTect Fast Bisulfite Conversion Kit (Qiagen, Hilden, Germany). In total, 250–500 ng of DNA were used as input.

### Methylation Microarray Analyses

To analyze genome-wide methylation, the Infinium MethylationEPIC BeadChip Kit (Illumina, San Diego, California, USA) was used according to the manufacturer’s instructions. Briefly, after bisulfite conversion, the DNA was amplified, enzymatically fragmented, and hybridized to microarray. The washed and stained microarray was analyzed on an iScan device (Illumina, San Diego, California, USA) to generate raw intensity (.idat) files.

### Bioinformatic Data Analysis and Statistics

Raw.idat files were imported into the latest version of R software (R Foundation for Statistical Computing, Vienna, Austria) for initial quality control and calculation of differential DNA methylation using the latest version of the RnBeads [16] package. Probes overlapping with SNPs, cross-reactive probes, and sex chromosome-specific probes were removed from further analyses. Low-quality probes were identified and removed using the Greedycut algorithm integrated in RnBeads. Data normalization was performed using the SWAN algorithm [17]. Hierarchical clustering was calculated based on all probes which passed the quality control. Calculation of methylation differences between groups was conducted using limma [18] as well as by computing a combined rank score, which depends on the difference in mean methylation levels of two groups, the mean methylation quotient and statistical significance. For subsequent analyses, the top 1000 differentially methylated CpG sites (DMPs) were selected. Gene Ontology (GO) and KEGG pathway enrichment analyses were performed using the missMethyl [19] package. Heatmaps were generated using the clustvis [20] package. The packages FactoMineR [21], factoextra [22], and umap [23] were used for dimensionality reduction analysis.

The clinical data were analyzed with the R programming language version 4.3.2. The distribution, central tendency, and dispersion of certain variables (categorical and quantitative) were analyzed to describe the patient population. Hypothesis testing was done with the appropriate statistical tests (e.g., Fisher’s exact test and log-rank test), and a two-tailed *p*-value below the significance level *α* = 0.05 was considered statistically significant. Survival analysis was performed with the Kaplan–Meier method using the R package ggsurvfit [24]. This study was exploratory and hypothesis-generating in nature.

## Results

### Patient Characteristics

A total of 54 tissue samples from 54 individual patients were collected at the Medical University of Vienna (*n* = 28) and at the Medical University of Graz (*n* = 26), comprising TC (*n* = 37, 68.5%) and AC (*n* = 17, 31.5%). Women were predominant in this cohort (64.8%), and the median age at diagnosis was 61 years (range 21–82). Most TC were diagnosed as stage 1 (78.4%), whereas most AC were stage 2 (29.4%) or higher (*p* = 0.002), see Table 1. Over the course of the disease, 25/53 (47.2%) developed metastases (TC vs. AC: *p* < 0.001), primarily to the liver (*n* = 19), bone (*n* = 12), brain (*n* = 7), and lungs (*n* = 7). SSTR imaging showed a positive scan in 14/26 patients (53.8%). Endocrine activity was present in 7/28 patients (25.0%). All tumors originated from the lung (primary lung NET). While most TC tissues (94.6%) were obtained from the lung, this was the case in only about half of the AC tissues (8/17). In total, 9/54 (16.7%) tissues were not from the initial diagnosis but were obtained later during the disease course (38–160 months).

**Table 1:** Patient demographics and basic disease characteristics

Variable	Typical carcinoid	Atypical carcinoid	Cohort overall
Number of patients (%)	37 (68.5%)	17 (31.5%)	54 (100%)
Sex			
Female	25 (67.6%)	10 (58.8%)	35 (64.8%)
Male	12 (32.4%)	7 (41.2%)	19 (35.2%)
Median age at diagnosis (range)	63 (21–82)	57 (34–76)	61 (21–82)
ECOG			
ECOG 0	13 (35.1%)	10 (58.8%)	23 (42.6%)
ECOG 1	0	1 (5.9%)	1 (1.9%)
Not available	24 (64.9%)	6 (35.3%)	30 (55.6%)
Primary tumor location			
Lung	37 (100%)	17 (100%)	54 (100%)
Tumor stage			
Stage 1	29 (78.4%)	2 (11.8%)	31 (57.4%)
Stage 2	3 (8.1%)	5 (29.4%)	8 (14.8%)
Other	5 (13.5%)	10 (58.8%)	15 (27.8%)
Ki-67 index			
Median	2	16.5	5
Not available	14	7	21
Metastasized at initial diagnosis			
Yes	3 (8.1%)	5 (29.4%)	8 (14.8%)
No	34 (91.9%)	12 (70.6%)	46 (85.2%)
Metastasized at any time during disease			
Yes	9 (24.3%)	16 (94.1%)	25 (48.3%)
No	27 (73.0%)	1 (5.9%)	28 (51.9%)
Not available	1 (2.7%)	0	1 (1.9%)
Endocrine activity			
Not available	21 (56.8%)	5 (29.4%)	26 (48.1%)
No	12 (32.4%)	9 (52.9%)	21 (38.9%)
Yes	4 (10.8%)	3 (17.6%)	7 (13.0%)
- Cushing syndrome	3	1	4
- Carcinoid syndrome	1	1	2
- Calcitonin-related	0	1	1
SSTR imaging			
Positive	11 (29.7%)	3 (17.6%)	14 (25.9%)
Mixed	0	6 (35.3%)	6 (11.1%)
Negative	3 (8.1%)	3 (17.6%)	6 (11.1%)
Not performed/not available	23 (62.2%)	5 (29.4%)	28 (51.9%)
Treatments (first line)			
Surgery	37 (100%)	13 (76.5%)	50 (92.6%)
Watch and wait	1	1	2
SSA	6	3	9
PRRT	3	1	4
Platin/etoposide	0	8	8
Everolimus	0	1	1
Other	0	1	1
Tissue sample origin			
Lung	35 (94.6%)	8 (47.1%)	43 (79.6%)
Liver	2 (5.4%)	5 (29.4%)	7 (13.0%)
Ovary	0	1 (5.9%)	1 (1.9%)
Lymph node	0	1 (5.9%)	1 (1.9%)
Not available	0	2 (11.8%)	2 (3.7%)
Tissue sample from diagnosis			
Yes	35 (94.6%)	10 (58.8%)	45 (83.3%)
No	2 (5.4%)	7 (41.2%)	9 (16.7%)

*ECOG*, Eastern Cooperative Oncology Group score; *SSTR*, somatostatin receptor; *SSA*, somatostatin analogs; *PRRT*, peptide receptor radionuclide therapy

In total, 50 patients (92.6%) had primary tumor resection. Surgery was not curative in 7 patients (for 2 no data was available), while 26 were recurrence-free at the last follow-up and 15 had a relapse (median time to relapse 47.4 months). The median overall survival (OS) of the entire patient cohort was 224.1 months (95% CI 116.9–not calculable) and the 10-year survival probability 69.0%. There was no difference in OS based on histology (median OS for TC not reached versus 161.1 months in AC, *p* = 0.6). Twenty-three patients (42.6%) started systemic therapy, with 5 being treated with adjuvant intent. The median progression-free survival (PFS) following systemic first-line therapy in the 18 patients with metastatic disease was 18.1 months (95% CI 6.0–27.7 months). The median PFS for the specific treatments was 5.4 (platinum/etoposide), 17.0 (everolimus), 14.5 (PRRT), 17.6 (other), and 23.6 months (somatostatin analogs).

### DNA Methylation in Typical Versus Atypical Lung NET

**Fig. 1:**
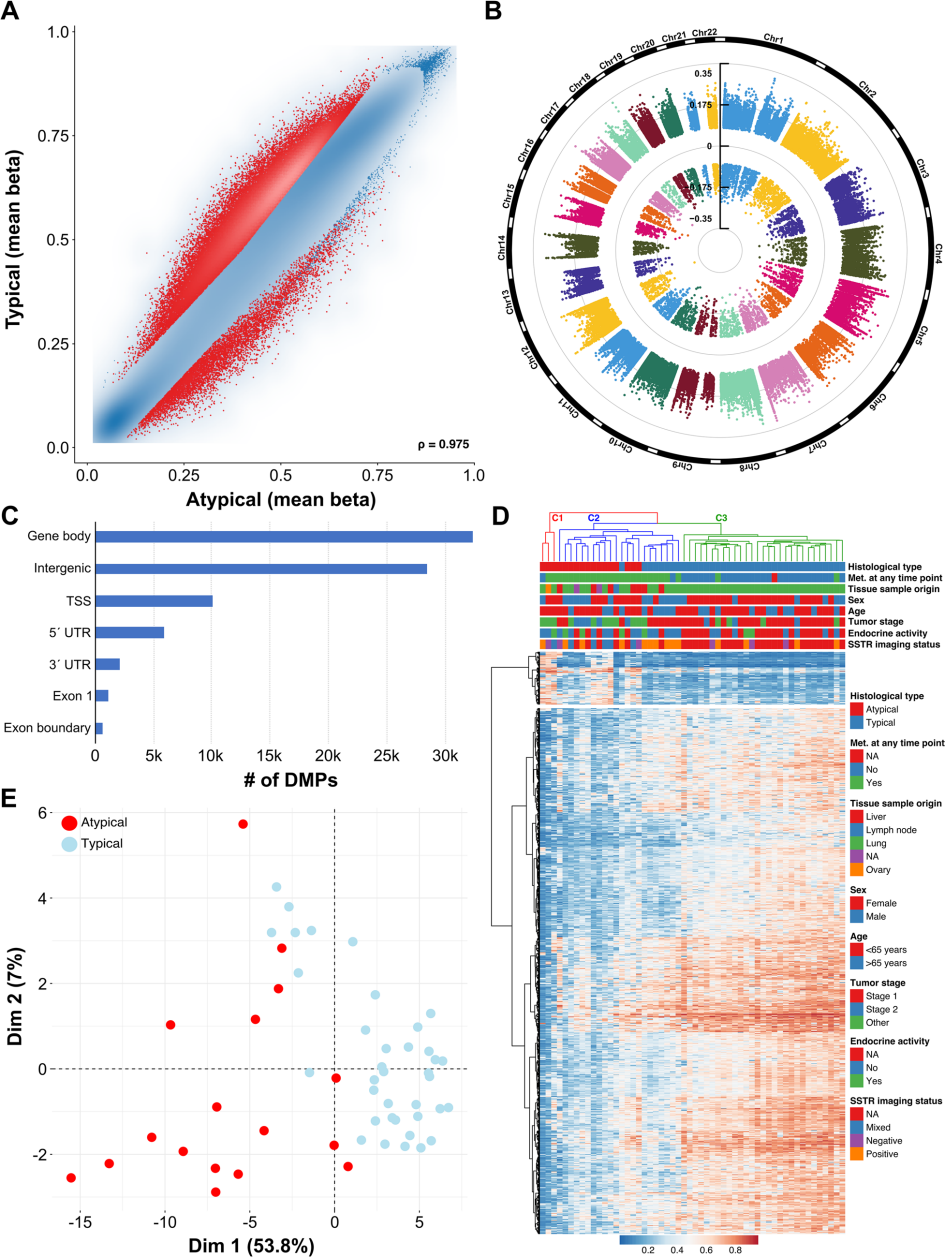
DNA methylation analysis of lung NET patients. **A** Scatter plot of differentially methylated CpG sites (DMPs) between typical carcinoid (TC) and atypical carcinoid (AC). Each dot represents a unique CpG site, and the red dots represent DMPs. **B** Circular Manhattan plot of the chromosomal distribution of these DMPs. **C** Genomic locations of DMPs (absolute figures in thousands). **D** Heatmap showing the hierarchical clustering based on the top 1000 DMPs between patients with TC and AC. **E** Unsupervised clustering using principal component analysis (based on total variance)

To characterize differences in the tumor methylomes within our lung NET cohort, we employed the Illumina MethylationEPIC BeadChip microarray technology. After quality control and probe filtering, 603.109 probes remained for further analysis. Differential methylation analyses between typical and atypical lung NET revealed substantial differences in both hypo- and hypermethylation (see Fig. 1A). These differentially methylated CpG probes (DMPs) were evenly spread over the chromosomes and were primarily located in gene bodies and in intergenic regions (40% and 35%, respectively, see Fig. 1B and C).

Hierarchical cluster analysis of the TC and AC samples using the topmost 1000 probes (909 hypomethylated and 91 hypermethylated in AC) identified three distinct subgroups, see Fig. 1D. The largest cluster C3 (right) included only typical carcinoids (*n* = 29, 100%), which were almost exclusively non-metastasized (*n* = 26/29, 89.7%), whereas cluster 2 (middle) was enriched with atypical carcinoids (*n* = 14/22, 63.6%) and consisted entirely of patients with metastatic disease except one case (*n* = 21/22, 95.5%). Based on the dendrogram in Fig. 1D, C1 was separated early from the two other clusters, suggesting that these three AC are more dissimilar from the C2/C3 tumors (see Discussion).

Furthermore, unsupervised clustering based on total variance was conducted using principal component analysis (PCA), see Fig. 1E. PC1 accounted for 53.8% of the variation in the data and PC2 for 7%. While TC samples clustered more tightly, AC samples showed greater variation in their methylome data. A similar pattern became evident in a UMAP (Uniform Manifold Approximation and Projection) graph, see Figure S1. Most typical carcinoids clustered separately from atypical carcinoids in the UMAP, indicating that they have a distinct methylation pattern.

### Potential Prognostic Role of Methylation Clusters

The identified clusters were examined for prognostic differences in PFS and OS. Only a few patients died during the follow-up period (*n* = 8), with two in the TC group and 6 in the AC subset. Hence, no clear OS difference was observed between TC and AC, see above. Consequently, the methylation clusters identified in Fig. 1D did not correspond to a statistically significant difference in prognosis, even though most events (*n* = 7) were recorded in cluster 2 (primarily atypical or metastatic carcinoids), with the median OS durations for C1 to C3 being 161.1, 224.1 months, and not reached, respectively, see Figure S2. In terms of therapies, everolimus was the most frequently applied drug (*n* = 10), but the survival results are restricted to a low number of patients (C1: *n* = 2, events = 2, median PFS 19.4; C2: *n* = 8, events = 6; median PFS 7.3 months; *p* = 0.8; C3: *n* = 0).

### Functional Classification of Methylation Differences Between TC and AC

**Fig. 2:**
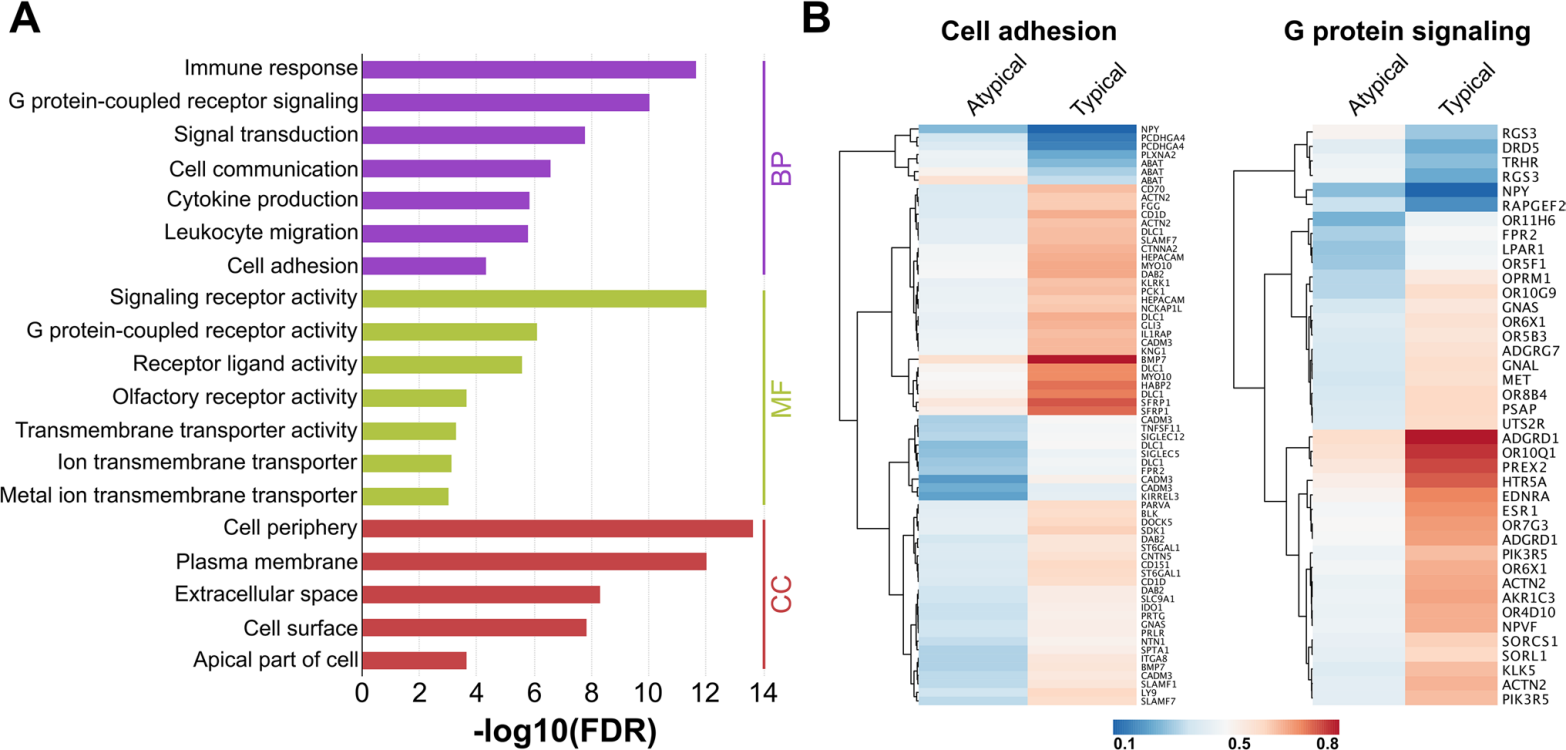
Functional classification. **A** Gene Ontology of the differentially methylated genes. FDR, false discovery rate; BP, biological process; MF, molecular function; CC, cellular component. **B** Heatmaps showing mean methylation of genes involved in G protein-coupled receptor signaling and cell adhesion in typical carcinoid (TC) and atypical carcinoid (AC) samples

For functional characterization of genes affected by differential methylation, DMPs located either 1500 bp around the transcription start site or in the first exon were subjected to Gene Ontology (GO) enrichment analyses. Figure 2A shows the GO categories that are most significantly enriched. Methylation differences were most significant within genes involved in immune response and G protein-coupled receptor signaling (biological processes, BP), signaling receptor activity (molecular functions, MF), and cell periphery and plasma membrane (cellular components, CC).

Therefore, it was of interest to further analyze the G protein-coupled receptor signaling pathway, which includes the SSTR encoding genes *SSTR1*, *SSTR2*, *SSTR3*, *SSTR4*, and *SSTR5*. Between TC and AC, several genes showed differential methylation in their promoter regions (see Fig. 2B); however, SSTR-encoding genes were not affected by differential methylation. For the GO category cell adhesion, differentially methylated promoters are also shown.

### Methylation Profiles According to Other NET Characteristics

#### Metastatic Cohort

Looking only at metastatic lung NET, typical and atypical carcinoids clustered separately (7/10 and 13/15 in the two clusters, respectively) and showed methylation differences, see Figure S3A. Likewise, considering only the TC cohort (Figure S3B, clustering based on samples with metastasized at any time point yes versus no), separation of the same cases (except one case) allocated to C2 (Fig. 1) was found.

#### SSTR Status

**Fig. 3:**
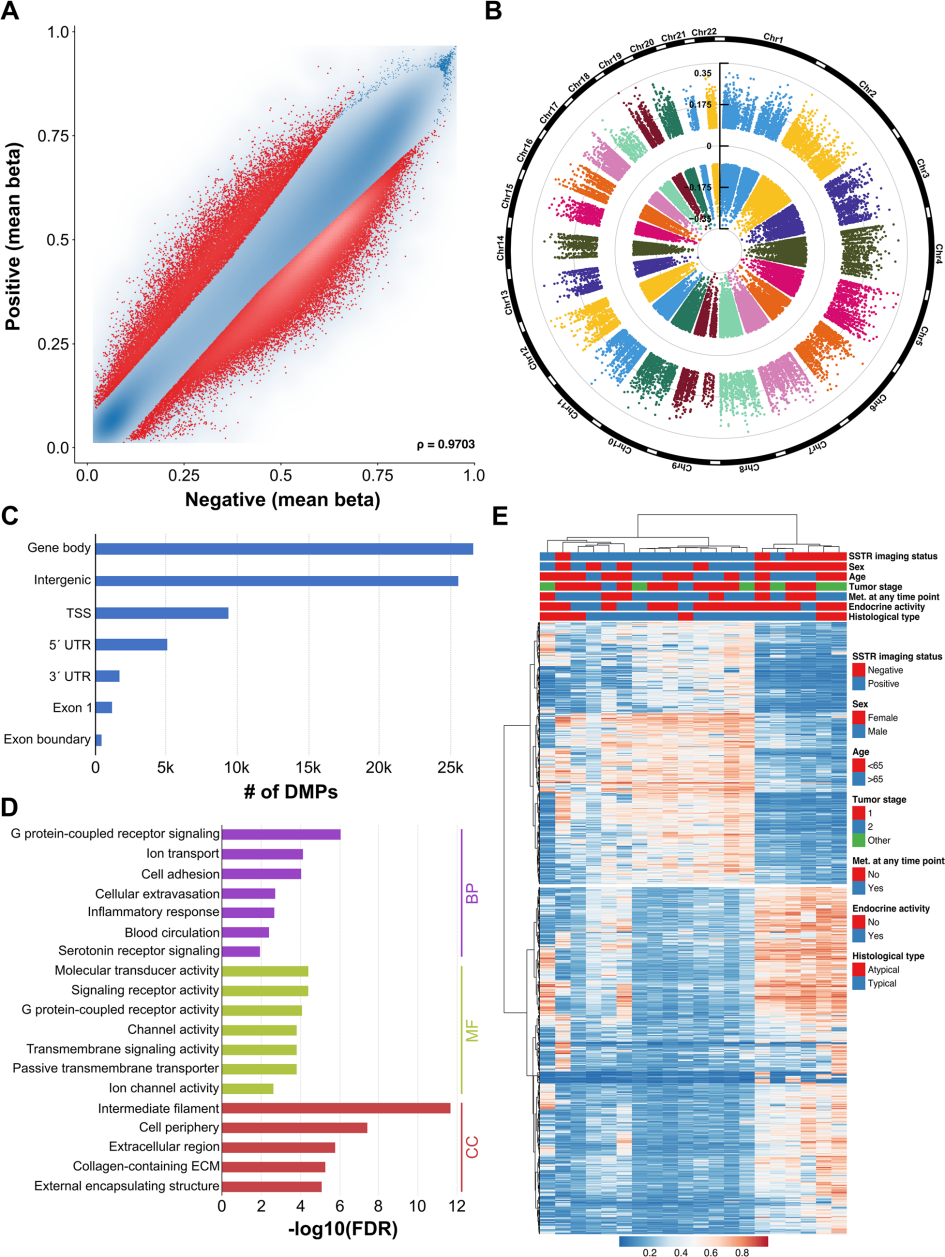
DNA methylation differences based on SSTR imaging status. **A** Scatter plot of DMPs between SSTR-positive and -negative patients. **B** Chromosomal distribution and **C** genomic locations of these DMPs. **D** Gene Ontology of genes that showed differential methylation. **E** Hierarchical clustering using the topmost 1000 DMPs

As shown in Fig. 3, methylation patterns of patients that were either positive or negative on SSTR imaging varied strongly. The differentially methylated CpG sites were regularly spread across the chromosomes and mostly located in gene bodies and intergenic regions, see Fig. 3B and C. In the cluster analysis using the top 1000 DMPs, we found that SSTR-negative tumors formed a separate methylation cluster (5/6 patients). Concordantly, several cell signaling GO categories were most significantly enriched, including G protein-coupled receptor signaling, serotonin receptor signaling, molecular transducer activity, and signaling receptor activity.

#### Endocrine Activity

**Fig. 4:**
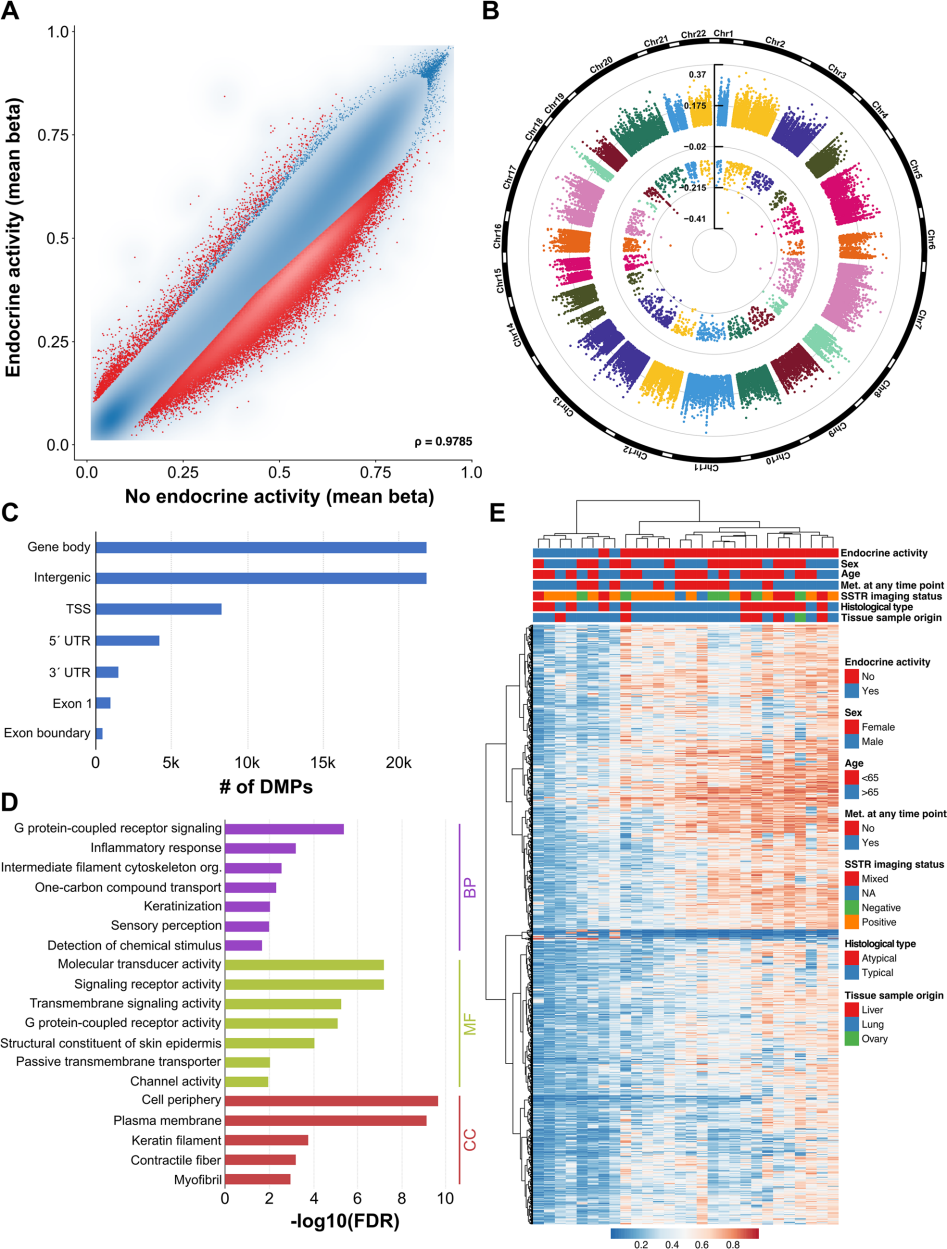
DNA methylation differences based on endocrine activity. **A** Scatter plot of DMPs between lung NET with versus without endocrine activity. **B** Chromosomal location and **C** genomic position of these DMPs. **D** Gene Ontology of the genes affected by differential methylation. **E** Clustering of samples based on the topmost 1000 DMPs

Similarly, methylation differences between tumors with versus without endocrine activity are shown in Fig. 4. Lung NET with no endocrine activity exhibited hypermethylation in the majority of differentially methylated CpG sites, while few were hypomethylated, see Fig. 4A. The chromosomal distribution and genomic location of these CpG sites were similar to previous analyses, see Fig. 4B and C. Hierarchical clustering suggested that hormonally active tumors have distinct methylation profiles, since they formed a distinct cluster (7/8 samples), see Fig. 4E. As previously, GO terms concerning cell signaling were implicated, see Fig. 4D.

## Discussion

Lung NET are rare tumors that are often sufficiently treated with curative surgery. Even in the metastatic setting, the prognosis can be good, particularly for TC. Histology plays an important part in determining the individual therapeutic approach, but further predictive and prognostic biomarkers are necessary. As previous research on DNA methylation in lung NET lacked detailed clinical characterization, we wanted to assess the DNA methylation profiles of known prognostic subgroups, i.e., histologic subtype, metastatic disease, SSTR2 status, and endocrine activity. Therefore, we collected tissue samples from 54 lung NET patients, with two-thirds having TC, half being metastasized, 7/28 having endocrine activity (25.0%), and 14/26 being SSTR imaging positive (53.8%).

In 2019, two groups published integrative multi-omics analyses of lung NET cohorts. Laddha et al. performed targeted DNA sequencing on 354 genes (*n* = 29), mRNA sequencing (*n* = 30), and methylation analysis using a 450K array (*n* = 18), and they could identify three distinct molecular subtypes based on gene expression, which were also consistent with the obtained DNA methylation data [14]. While the clinical information was limited to radiological-pathological data, tissues in cluster 1 were shown to be predominantly from female patients and were located in the peripheral lung, cluster 3 tumors were mainly found at an endobronchial location and obtained from younger patients, and MEN1 mutations were enriched exclusively in cluster 2 [14]. Interestingly, no gene expression differences were observed between TC versus AC in this study [14]. Alcala et al. included 257 lung NEN (81 TC, 35 AC, 75 LCNEC, and 66 SCLC) in their integrative machine-learning-based study, using an 850K array for the epigenome analysis of 95 samples [15]. Based on Multi-Omics Factor Analysis and consensus clustering (transcriptome and methylome data), they could identify three clusters enriched for distinct tumor subtypes, i.e., one cluster included 75% TC, another 54% AC, and the third 92% of all LCNEC [15].

In our analysis, we found different methylation patterns for specific pathologic characteristics, that is, histologic type and metastatic disease. C3 was 100% TC and about 90% non-metastatic, whereas C2 consisted of two-thirds AC and more than 95% metastasized tumors. Notably, previously implicated clinical factors (Laddha et al.) [14] such as being female (45% in C2 and 79% in C3 of our analysis) and young age (< 65 years, 59% versus 66%) did not clearly segregate here. MEN1 and other genomic alterations were not investigated and cannot be addressed in this study. Moreover, as with the results from Alcala et al. [15], there was a mixed group of TC and AC, suggesting that DNA methylation-based clustering analyses alone might not be able to make accurate pathologic diagnoses. Inversely, one could also surmise that current pathologic criteria (e.g., morphological growth pattern, cytological features, mitotic count, and presence of necrosis) [2] are insufficient for optimal classification of lung NET, and that DNA methylation could potentially provide additional information, as is the case for the classification of central nervous system tumors [25].

The sample NET12 deserves further discussion, as the patient had a TC and additionally a pathological diagnosis of diffuse idiopathic pulmonary neuroendocrine cell hyperplasia (DIPNECH) in the contralateral lung. Formally, these are considered preneoplastic changes, but they may be difficult to distinguish from metastatic disease in case of multiple lesions. In this case, it was initially assumed that the patient had pulmonary metastases, so treatment with lanreotide was administered. Interestingly, the cluster analysis here supports the notion that the TC analyzed in this patient is non-metastatic, since it was only one of two samples in C3 recorded as having metastatic disease, and these multifocal lesions therefore appear to be independent. This shows that DNA methylation analyses might be able to indicate certain clinical characteristics.

Furthermore, the C1 cluster (*n* = 3) included the two AC cases with the highest Ki-67 index observed in this cohort (NET51 and NET20 both had a Ki-67 of 30%), while in C2, all AC had a Ki-67 < 21%. These two lung NET samples seem to resemble the highly proliferating NET G3 cases of gastroenteropancreatic origin and might relate to the recently recognized entity of “carcinoids with elevated mitotic counts and/or Ki67 proliferation index” [2] or to the discovery of supra-carcinoids (tumors with carcinoid morphology but LC-NEC molecular characteristics) in the methylation study from Alcala et al. [15]. Therefore, introducing a WHO NET G3 category also for lung NET might be a solution for the better categorization of these cases. Confirming and further characterizing this particular cluster is of great interest; thus, we aim to collect such cases for further investigation.

Given the indolent behavior of many lung carcinoids and the curative-intent treatment in the majority of our patients, we could not observe any prognostic difference based on the DNA methylation clusters. However, histologic type and metastatic state are known prognostic factors that translate into a survival difference in larger collectives [6].

Moreover, we analyzed DNA methylation patterns according to somatostatin receptor (SSTR) imaging status. SSTR expression is the main rationale for somatostatin analog treatment in lung NET, and SSTR assessment by immunohistochemistry or imaging is recommended by guidelines before therapy starts [10]. The expression of SSTR and of somatostatin is epigenetically regulated [26]. Here, we demonstrated that there are differentially methylated CpG sites between SSTR-positive and -negative lung NET. However, in the pathway analysis (Fig. 2B), the promoter regions of the somatostatin receptors did not show differential methylation, suggesting that other epigenetic differences between TC and AC are involved.

Finally, a certain fraction of lung NET patients show specific hormonal syndromes which is estimated at around 8% for carcinoid syndrome [5] or < 5% for Cushing syndrome [27]. Without treatment, mortality can be high in functioning NET due to possible complications [28]. In the clustering analysis according to endocrine activity, NET with endocrine activity got enriched in one of two clusters. Methylation analysis could therefore provide an indication of patients who should potentially be assessed more closely for subclinical endocrine syndromes.

Given the key role of epigenetics in lung NET, epigenetically active substances could be used in the future; initial studies are already underway or have been completed with mixed results in certain cases [29].

There are, however, several limitations to our analysis. First, due to the rarity of lung NET, tissue availability is limited, so we could not be too restrictive by excluding patients who lacked certain clinical features, and no information on RNA sequencing or *MEN1* mutation status was available. Second, not all tissues included were from the initial diagnosis and from the primary tumor in the lung. To the best of our knowledge, it is unclear whether methylation patterns change significantly during the disease course and development of metastases.

Taken together, we have assembled one of the largest methylation cohorts of lung NET to date in order to integrate biological tumor characteristics with clinical information, allowing us to characterize the methylation patterns of TC and AC, and to demonstrate methylation differences between metastasized versus non-metastasized lung NET as well as differences between SSTR imaging positive versus negative tumors and hormonally active versus inactive tumors. Overall, our comprehensive analysis supports that methylation profiling is a helpful tool that should be integrated in prospective studies.

## Supplementary Information

Below is the link to the electronic supplementary material.

Supplementary file1 UMAP (Uniform Manifold Approximation and Projection) graph for all typical and atypical carcinoid samples. (TIFF 1135 KB)

Supplementary file2 Kaplan–Meier plot of overall survival (OS) according to the clusters identified in Figure 1D. (TIFF 2438 KB)

Supplementary file3 Heatmap showing clustering between typical and atypical carcinoids (only metastatic) (A) and between typical carcinoids with versus without metastases at any time point (B). (TIFF 4802 KB)

## Data Availability

Raw data of the DNA methylation analysis as well as further data supporting the findings of this study can be made available upon reasonable request to the corresponding author.
